# Syndrome Sinistre: Left Brachiocephalic Vein Compression and its Neurological Manifestations

**DOI:** 10.3390/neurolint16050087

**Published:** 2024-10-17

**Authors:** Karthikeyan M. Arcot, Vincent S. DeOrchis

**Affiliations:** 1 St. Francis Hospital & Heart Center 100 Port Washington Blvd, Roslyn, NY 11576, USA; vsdeorchis@marinelex.com; 2 Department of Neurology, New York University Langone Hospital Long Island 259 First St, Mineola, NY 11501, USA

**Keywords:** venous outflow obstruction disorder, brachiocephalic vein compression, neurodegeneration, venous congestion, headache, brain fog

## Abstract

Embryologically, the left brachiocephalic vein (LBV) originates as an anastomotic channel between the right and left anterior cardinal veins. This positions the LBV between the manubrium sterni anteriorly and the innominate artery posteriorly. This pattern of adjacency of the aorta to the LBV is unique to mammals and results from a quirk of evolution. With age, the ascending aorta unfolds, elongates and dilates. Simultaneously, there is a change in the thoracic geometry that reduces the thoracic volume primarily from disc height loss and kyphosis. These transitions progressively compress the LBV. Normally, this compression is circumvented via collateral pathways and “Blood finds a way”. However, traversing these circuitous pathways comes at a cost and can result in delayed transit times and venous congestion. While it is possible that compression of the LBV in the setting of adequate collateral channels may fail to provoke any pathologic sequelae, we propose a phenomenon in which such compression in the setting of inadequate collateral circulation may lead to a state of pathologic venous congestion. This anatomic anomaly and its associated clinical features, if identified, can offer a new avenue for treatment options for some of the hitherto unexplained neurologic disorders.

## 1. Background

Embryologically, the left brachiocephalic vein (LBV) originates as an anastomotic channel between the right and left anterior cardinal veins [1]. This positions the LBV between the manubrium sterni anteriorly and the innominate artery posteriorly [2]. This pattern of adjacency of the aorta to the LBV is unique to mammals and results from a quirk of evolution [3]. With age, the ascending aorta unfolds, elongates and dilates [4]. Simultaneously, there is a change in the thoracic geometry that reduces the thoracic volume primarily from disc height loss and kyphosis [5]. These transitions progressively compress the LBV. Normally, this compression is circumvented via collateral pathways and “Blood finds a way” [6]. However, traversing these circuitous pathways comes at a cost and can result in delayed transit times and venous congestion. While it is possible that compression of the LBV in the setting of adequate collateral channels may fail to provoke any pathologic sequelae, we propose a phenomenon in which such compression in the setting of inadequate collateral circulation may lead to a state of pathologic venous congestion. This anatomic anomaly and its associated clinical features, if identified, can offer a new avenue for treatment options for some of the hitherto unexplained neurologic disorders.

## 2. Embryology, Anatomy, Radiology and Clinical Features

The anterior cardinal veins drain the cephalic portion of the embryo. During week eight of development, the thyroid and thymic veins join to form a large transverse anastomosis superior to the common cardinal veins. This anastomosis allows blood from the cephalic portion of the left anterior cardinal vein (future left internal jugular vein) to reach the junction of the right anterior cardinal vein (future right internal jugular vein) and right common cardinal vein (future superior vena cava (SVC)). Various developmental venous malformations (left-sided SVC, double SVC) may occur if the above does not proceed according to plan [7]. However, normally, one ends up with an efficient LBV that transports blood from the left internal jugular vein to the superior vena cava, until, of course, it is smothered by its sturdier neighbors.

The LBV vein is approximately 6 cm to 7 cm long. It is formed by the confluence of the left internal jugular vein and the left subclavian vein behind the left sternoclavicular joint. It then runs an oblique and downward course to the right to join the right brachiocephalic vein and form the superior vena cava behind the sternum and the first intercostal space. In its course, it receives the following veins: left vertebral, left inferior thyroid, left internal thoracic, left superior intercostal, left supreme intercostal, thymic and pericardiacophrenic veins. Its relationship with its less compliant neighbors forms the crux of this syndrome. It initially runs anterior to the parietal pleura of the lung and then enters a valley between the bony manubrium anteriorly and an unyielding innominate artery posteriorly [2]. It is in this valley that the LBV becomes entrapped, usually for a few millimeters congruent with the portion in contact with the innominate artery.

**Figure 1:**
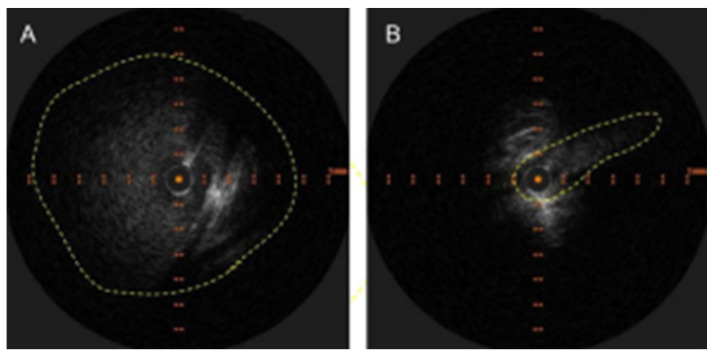
(**A**) Appearance of LBV on intravascular ultrasound with manual demarcation of the approximate border with dashed yellow lines. (**B**) Severe slit-like compression of LBV.

The radiological findings are often obvious, but the apparent lack of clinical relevance lends to underreporting. As an example, osteoporotic vertebral fractures suffer a similar fate [8]. There are excellent examples of LBV compression with resultant venous reflux on CT angiography [9,10]. Similar findings are noted in MR venography [11]. However, this condition will continue to elude detection by CT angiography if right-sided injections are performed [10] and MR venography of the neck if coverage does not extend down to the level of the LBV. Intravascular ultrasound (IVUS) should be the new “gold standard” for diagnosis as it clearly identifies and demonstrates the dramatic decrease in luminal area while providing accurate vessel measurements that are necessary for treatment (Figure 1).

The clinical presentation of this condition can range from being completely asymptomatic to a combination of headaches, often chronic, exacerbated by exertion or valsalva, and frequently refractory to standard medical treatment, vertigo, dysequilibrium, tinnitus or hearing impairment. Some patients report a disabling “brain fog” in addition.

**Figure 2:**
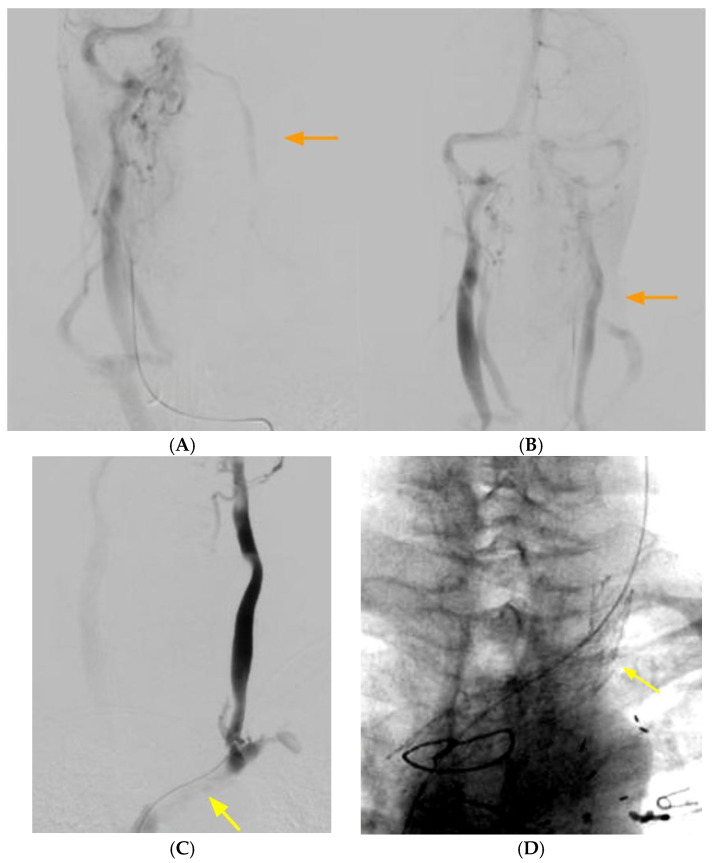
(**A**) Delayed phase of arterial angiogram showing poor flow in the left internal jugular vein (orange arrow) with (**B**) normalization after stenting of the left brachiocephalic vein (orange arrow). (**C**) Left internal jugular vein injection with retrograde flow into the cranium, continuing across the torcula and emptying via the right internal jugular vein due to LBV compression (yellow arrow). (**D**) Left brachiocephalic stent (yellow arrow).

Conventional catheter-based angiography is useful in depicting the delay in venous emptying or stagnation or slowing of flow in the left internal jugular vein. It is also useful in depicting a radiological improvement in flow after treatment (Figure 2). Conventional catheter-based venography is a poor modality to detect this condition, as the plane of compression is usually parallel to the anteroposterior plane, and the lateral plane is obscured by the patient’s arms. Venous stagnation, reflux and diverticula are often noted.

**Figure 3:**
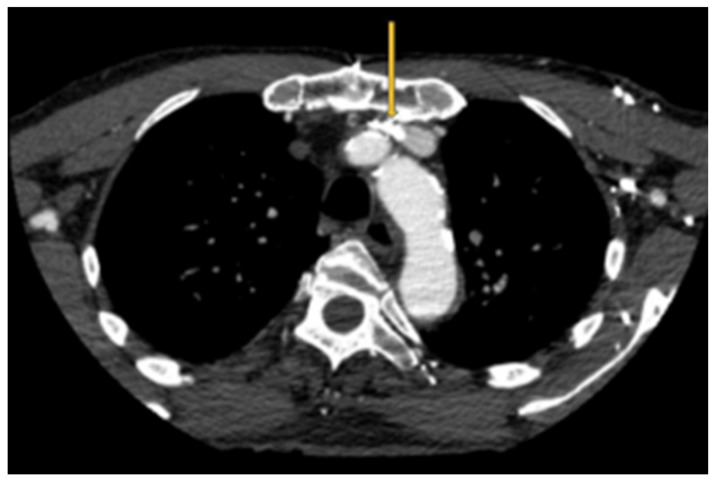
Pacemaker leads (orange arrow) compounding the venous outflow obstruction in the setting of LBV compression.

As an interesting aside, an intersection between pacemakers/defibrillators and LBV compression occurs. The leads are typically inserted on the side of the non-dominant hand [12]. They can occupy some valuable space in the LBV and produce the above symptoms (Figure 3). Future research focused on this entity can help cardiac electrophysiologists detect this anomaly and opt for right-sided insertion, leadless pacemakers or subcutaneous defibrillators. Treatment options that need to be studied include exercises aimed at improving venous collaterals, posture rehabilitation and stenting of the left brachiocephalic vein.

## 3. Discussion

LBV compression should be expected to occur and progress with aging due to the anatomical factors described above. It should generally be asymptomatic given the remarkable redundancy of the venous system and its collaterals. Symptoms should and do ensue when the venous collaterals are either insufficient or overwhelmed. This can be compounded by venous obstruction on the contralateral side, which is not uncommon. For example, trapping of the right internal jugular vein at the level of the C1 transverse process in a patient with pre-existing LBV narrowing, both of which occur as a result of age-related changes, may provoke a state of cerebral venous congestion as outflow collateral pathways are limited. In fact, bilateral outflow obstruction might be a necessary condition, given the robust nature of the connections between the dural sinuses and the remarkable venous collaterals in the cervical region. This results in the emergence of two pathophysiological states. One is governed by a buildup of pressure due to venous hypertension and the other is governed by stagnation/venous congestion. The venous hypertensive state could potentially explain the physical effects such as headache and obstruction of cerebrospinal fluid outflow and the resultant clinical conditions. Accumulation of toxic waste metabolites [13] due to poor venous outflow could potentially be a risk factor for the development of some neurodegenerative conditions. Aging is a risk factor for neurodegenerative disease [14] and aging is a risk factor for LBV compression. There have been multiple attempts to link singular neurological disorders to venous outflow obstruction. Idiopathic intracranial hypertension, aka pseudotumor cerebri, has often been attributed to “dysfunction of arachnoid granulations” resulting in poor CSF clearance. Data supporting the presence of transverse sinus stenosis as a structural risk factor, compounded by obesity, have been given much more attention and are now often quoted in articles on the topic [15]. Additionally, the association of venous outflow insufficiency with other conditions such as transient global amnesia [11], deafness [16] and normal pressure hydrocephalus [17] are now being investigated.

One could argue that a more holistic approach is in order. If such an approach was applied, the entire class of disorders, neurological or otherwise, would come under an umbrella term such as Venous Outflow Obstruction Disorders (VOODO). The venous hierarchy (for the superior vena caval system) would be along anatomical lines beginning with the cortical veins, coursing through the superior sagittal, inferior sagittal and straight sinuses, moving on to the transverse and sigmoid sinuses, followed by the paired jugular and brachiocephalic veins with the superior vena cava and the right heart and pulmonary arteries forming the inferior portions. Theoretically, any downstream segment in this hierarchy should be able to affect any upstream segment and produce a disorder based on the affected venous territory. For example, one should not be surprised when tandem stenoses involving the superior vena cava and transverse sinus produce papilledema in a patient and similarly not be surprised if transverse sinus stenting alone does not relieve the papilledema in said patient. Hence, it would be important to evaluate the entire pathway. The treatment should address tandem and bilateral obstructions with the aim of restoring good venous outflow, from both the superficial and deep pathways. LBV compression would fall into this class of disorders. The symptoms would depend on the affected upstream pathway and the area of the nervous system or organ that it drains. On the surface, this condition appears to be associated with seemingly innocuous complaints such as headache and vertigo. At a deeper level, it may precede more sinister neurological and neurodegenerative disorders. Given the simplicity of its treatment with stenting, it represents a serious target for further research along with other venous outflow obstruction disorders.

## Data Availability

The data presented in this study are openly available in preprints.org at 10.20944/preprints202307.0077.v1, reference number 2023070077.
